# Supplementary material for: Enhanced diffusion by binding to the crosslinks of a polymer gel
Source: Nat Commun. 2018 Oct 19;9:4348. doi: 10.1038/s41467-018-06851-5 (PMC6195553; doi:10.1038/s41467-018-06851-5)
Supplement: Supplementary file 2 — Description of Additional Supplementary Files [file 41467_2018_6851_MOESM2_ESM.pdf]

Description of Additional Supplementary Files

File Name: Supplementary Movie 1
Description: Brownian Dynamics simulation of perfect filtering in a gel with high binding affinities. The interactions between the binding particle (blue), non-binding particle (red) and gel (green) are described in the text. The movie shows that the binding particle is able to slip past the crosslinks via the mechanism shown in Fig. 3C of the main text, allowing it to diffuse through the gel. The binding particle, however, remains caged and does not diffuse. The blue and red lines show the trajectory history for both particles.

File Name: Supplementary Movie 2
Description: Demonstration of a perfect filter in the limit of high binding affinity. Initially, a gel is trimmed to form a circular shell. 10 binding (blue) and 10 non-binding (red) particles are all placed within this shell. Over time, the binding particles are able to enter the gel and diffuse through it, while the nonbinding particles remain in the interior. Note that around 7 seconds into the movie, two binding particles on the right-hand side rearrange the gel topology, resulting in one of the particles becoming stuck. This movie corresponds to Fig. 4B in the main text.

File Name: Supplementary Movie 3
Description: Brownian Dynamics simulation of enhanced diffusion in a gel with moderate binding affinities. The interactions between the binding particle (blue), non-binding particle (red) and gel (green) are described in the text. The blue and red lines show the trajectory history for both particles. In the limit of moderate binding affinity, both binding and non-binding particles are able to diffuse. While it is not obvious from the movie which particle diffuses faster, Fig. 5C in the main text shows that the binding particle has a significantly larger diffusion constant.
